# Allosteric Modulators of Serotonin Receptors: A Medicinal Chemistry Survey

**DOI:** 10.3390/ph17060695

**Published:** 2024-05-28

**Authors:** Leonardo Brunetti, Fabio Francavilla, Marcello Leopoldo, Enza Lacivita

**Affiliations:** Department of Pharmacy–Drug Sciences, University of Bari Aldo Moro, 70125 Bari, Italy; leonardo.brunetti@uniba.it (L.B.); fabio.francavilla@uniba.it (F.F.); enza.lacivita@uniba.it (E.L.)

**Keywords:** serotonin, GPCR, natural compounds, allosteric modulators, cannabinoids

## Abstract

Serotonin (5-hydroxytryptamine, 5-HT) is a neurotransmitter regulating numerous physiological functions, and its dysregulation is a crucial component of the pathological processes of schizophrenia, depression, migraines, and obesity. 5-HT interacts with 14 different receptors, of which 5-HT_1A-1F_Rs, 5-HT_2A-C_Rs, and 5-HT_4-7_Rs are G protein-coupled receptors (GPCRs), while 5-HT_3_R is a ligand-gated ion channel. Over the years, selective orthosteric ligands have been identified for almost all serotonin receptors, yielding several clinically relevant drugs. However, the high degree of homology between 5-HTRs and other GPCRs means that orthosteric ligands can have severe side effects. Thus, there has recently been increased interest in developing safer ligands of GPCRs, which bind to less conserved, more specific sites, distinct from that of the receptor’s natural ligand. The present review describes the identification of allosteric ligands of serotonin receptors, which are largely natural compounds (oleamide, cannabidiol, THC, and aporphine alkaloids), complemented by synthetic modulators developed in large part for the 5-HT_2C_ receptor. The latter are positive allosteric modulators sought after for their potential as drugs preferable over the orthosteric agonists as antiobesity agents for their potentially safer profile. When available, details on the interactions between the ligand and allosteric binding site will be provided. An outlook on future research in the field will also be provided.

## 1. Introduction

The concept of allosteric modulation for enzymes was first introduced with the Monod–Wyman–Changeux model [1]. Subsequently, this concept was expanded to other types of proteins, including receptors [2]. Nowadays, the design of allosteric modulators is a consolidated strategy for the development of novel pharmaceutics. According to the Allosteric Database, there are a total of 68 clinical candidates in Phase I (23), Phase II (36), and Phase III (9) studies and 19 FDA-approved allosteric drugs, of which 4 target GABA-A receptor, 2 AMPA receptor, and only 1 a GPCR (the extracellular calcium-sensing receptor) [3].

An allosteric modulator and an orthosteric agonist bind concomitantly to the target receptor, forming a ternary complex in which the allosteric modulator influences the agonist’s binding affinity and operational efficacy [4].

The binding of the orthosteric ligand to a site separate from the site of the allosteric modulator leads to a permissive system (i.e., the action of one may not necessarily preclude the action of the other). As a consequence, there are various categories of allosteric modulators: negative allosteric modulators (NAMs), positive allosteric modulators (PAMs), PAM-antagonists, NAM-agonists, and PAM-agonists [5,6,7]. Somewhat less frequently, allosteric inverse agonists have also been described in the literature, with their allosteric action being targeted to second messengers further down the line of signal transduction [8,9].

NAMs decrease the receptor´s response to orthosteric agonists, with a rightward shift of the dose–response curve and/or a reduction in maximal response. PAMs, inversely, potentiate the response to orthosteric agonists, causing a leftward shift of the dose–response curves and/or an increase in maximal response. PAM-antagonists are allosteric modulators that increase the affinity but decrease the efficacy of agonists, producing a leftward shift of the dose–response curve and a reduction in the maximal response. NAM-agonists produce NAM effects in combination with direct agonism. PAM-agonists produce PAM effects with concomitant direct agonism. Finally, silent/neutral allosteric modulators can bind to the allosteric site and prevent the binding of other allosteric ligands without changing the affinity or efficacy of the orthosteric agonists [10]. One notable example is flumazenil, which reverses the effects of benzodiazepines by competitive inhibition at the benzodiazepine recognition site on the GABA A receptor, thus being an antidote in case of benzodiazepine overdose [11].

Other excellent reviews regarding the details of the pharmacological mechanisms of allosteric modulators are available for readers wishing for more comprehensive sources of information on this topic [4,10].

G protein-coupled receptors (GPCRs) belong to the largest family of transmembrane proteins and regulate a wide range of physiological processes in response to signals coming from outside the cell. These receptors have proven to be the most successful class of drug targets, with more than 700 drugs approved by the FDA, targeting 134 GPCRs [12].

Over the years, increasing evidence has shown the importance of multiple signal transduction pathways associated with a single receptor (i.e., different effector G proteins and β-arrestins), bringing to light numerous opportunities for developing new drugs. In parallel, the identification of ligands that target allosteric binding sites is attracting scientific interest with the promise that such allosteric modulators may offer new opportunities to design GPCR-targeting therapeutics. In fact, even though GPCRs remain some of the most promising targets for novel pharmaceuticals, the probability of a new drug reaching the market is low, as >90% of the compounds entering Phase I clinical trials fail to achieve FDA approval, mostly because of lack of efficacy or safety concerns [13].

In this context, the design of allosteric modulators appears to be an innovative targeting approach that can distinguish between highly homologous receptor subtypes by exploiting less evolutionarily conserved allosteric sites, leading to improved selectivity and, ultimately, safety [14].

**Figure 1:**
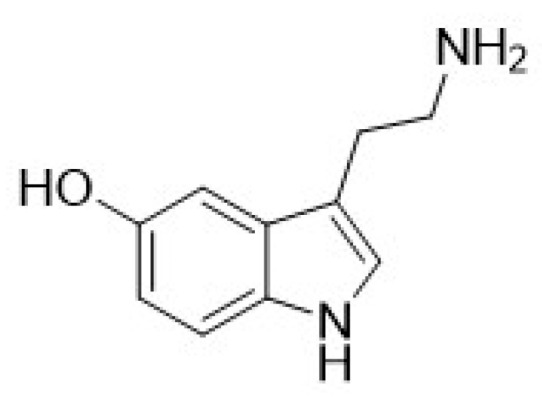
Structural formula of serotonin.

Serotonin (Figure 1), or 5-hydroxytryptamine (5-HT), is a neurotransmitter implicated in various physiological processes, including sleep–wake cycles, memory, cognition, mood regulation, anxiety, emesis, appetite, gut motility, blood clotting, breathing, and many others [15]. Dysregulation of the serotonin system often leads to pathological conditions [16].

The serotoninergic system has been extensively studied and has been the subject of many excellent and comprehensive reviews. For in-depth details, the reader is referred to Refs. [17,18,19]. In Table 1 are listed information related to the key characteristics of serotonin receptors, including signaling, distribution, and physio-pathological role.

Serotonin exerts its effects through interactions with its receptors (5-HTRs), a family that comprises twelve G protein-coupled receptors (GPCRs) and one ligand-gated cation channel [17].

The metabotropic 5-HTRs belong to the “type A” (rhodopsin-like) family of GPCRs and feature the typical structure, which includes seven transmembrane (7-TM) helices with hydrophobic regions, three intracellular domains, and three extracellular domains. The N-terminus is located in extracellular space, while the C-terminus is in intracellular space [20]. G protein-coupled 5-HT receptors can interact with three distinct types of G proteins: G_i/o_, which inhibits adenylyl cyclase (AC), leading to an intracellular reduction in cyclic adenosine monophosphate (cAMP) concentration; G_s_, which activates AC, resulting in an elevation of cAMP concentration; and G_q/11_, which activates phospholipase C, inducing an increase in inositol triphosphate (IP3), diacylglycerol (DAG), and calcium ion (Ca^2+^) concentrations. In addition, 5-HTRs may signal through recruitment of beta-arrestin 2 [18,21].

Specifically, 5-HT_1A_R, 5-HT_1B_R, 5-HT_1D_R, 5-HT_1E_R, and 5-HT_5_R couple with G_i/o_; 5-HT_4_R, 5-HT_6_R, and 5-HT_7_R couple with a G_s_; and 5-HT_2A_-CRs couple with G_q/11_ [22]. Furthermore, it has been noted that 5-HT_4_R can couple with G_s_ or G_i_, with a preference for the former [23], while 5-HT_7_R can also couple with G_12_ [24].

The 5-HT_1_R subfamily includes 5-HT_1A_, 5-HT_1B_, 5-HT_1D_, 5-HT_1E_, and 5-HT_1F_ receptors, all of which are coupled with G_i/o_ proteins. 5-HT_1A_R functions as both a presynaptic receptor in the raphe nuclei, where it regulates 5-HT release in the CNS, and as a postsynaptic receptor in the medial prefrontal cortex and other cortical areas, modulating dopamine release [25]. 5-HT_1A_R modulation extends to various brain functions such as mood, anxiety, cognition, sleep, and pain perception [26]. 5-HT_1B,D,F_Rs are implicated in the pathophysiology of migraines and exhibit differential expression across various regions [15]. Specifically, 5-HT_1B_R is predominantly found in the CNS and peripheral vasculature and shares high sequence homology with 5-HT_1D_R. 5-HT_1E_R is mainly expressed in the hippocampus and hypothalamus, and 5-HT_1F_R is prevalent in cortical and hippocampal areas [16].

The 5-HT_2_Rs subfamily encompasses 5-HT_2A_R, 5-HT_2B_R, and 5-HT_2C_R that couple with G_q/11_ [22]. These receptors are widely distributed throughout the brain and peripheral tissues. Specifically, 5-HT_2A_R contributes to regulating vascular tone, mood, cognition, and hallucinogenic effects of various alkaloids [27]. 5-HT_2B_R is predominantly found in smooth muscle cells, cardiac tissue, and endothelial cells, playing a crucial role in cardiovascular function. 5-HT_2C_R is present in the gastrointestinal tract, where it participates in regulating gut motility and appetite [28], and is abundantly expressed in the CNS, where it is responsible for many of 5-HT´s central effects on appetite, cognition, mood, and other functions. This receptor is a significant pharmacological target for addiction, anxiety, depression, epilepsy, schizophrenia, and obesity, all conditions in which it is involved [17].

The 5-HT_3_R belongs to the Cys-loop family of ligand-gated ion channels and functions as a selective cation channel. It is composed of five symmetrically arranged subunits that encircle a central ion-conducting pore [29]. Multiple subunits of 5-HT_3_R exist, with subunits A and B being the most extensively studied, though others, such as C, D, or E, have also been described. These subunits can polymerize, leading to the formation of either a homomeric variant of the channel, such as 5-HT_3A_R or 5-HT_3B_R, or heteromeric configurations, such as 5-HT_3A/B_R [30]. In the CNS, 5-HT_3_R is distributed across several regions (cortex, hippocampus, nucleus accumbens, substantia nigra, and ventral tegmental area) where it modulates processes such as emesis, cognition, and anxiety. Notably, high levels of this receptor are observed in the brain stem, particularly in areas associated with the vomiting reflex, such as the area postrema and nucleus tractus solitarius. In the peripheral nervous system (PNS), 5-HT_3_R is involved in various sympathetic, parasympathetic, and sensory functions, particularly in regulating peristaltic activity and urinary function [30].

5-HT_4_R, 5-HT_6_R, and 5-HT_7_R are all coupled with a G_s_. 5-HT_4_R, which is expressed in various regions of the CNS and also in peripheral tissues, including the heart and gastrointestinal tract, where it modulates motility, secretion, and visceral sensitivity [31]. 5-HT_6_R and 5-HT_7_R, on the other hand, are expressed mainly in the CNS. 5-HT_6_R has been mainly linked to learning and memory, addiction, and seizure control [18]. 5-HT_7_R is especially represented in the spinal cord, hippocampus, and thalamus, where it regulates cognitive functions [32], circadian rhythm, and mood [24].

Information regarding 5-HT_5_R, which, like 5-HT_1_R, couples with G_i/o_, is much more limited. Two isoforms have been described, 5-HT_5A_R and 5-HT_5B_R, although only the former receptor is expressed in humans. The gene for 5-HT_5B_R is present in the human genome; however, its coding sequence is interrupted by stop codons, suggesting that it was lost during human evolution [33]. 5-HT_5A_R receptors are primarily located in brain regions associated with memory and learning, where they contribute to neurotransmitter release and synaptic plasticity [34].

**Table 1:** Summary of 5-HT receptor subtypes, their distributions, and their physio-pathological implications.

Receptor	G Protein/Type of Ion Channel	Distribution	Physio-Pathological Implications	Refs.
5-HT_1A_R	G_i/o_	Raphe nuclei (presynaptic receptor), hippocampus, prefrontal cortex, thalamus, lateral septum, amygdala, hypothalamic nuclei (postsynaptic receptor)	Modulation of mood, anxiety, cognition, sleep, and pain perception	[25,26]
5-HT_1B_R	G_i/o_	Substantia nigra, globus pallidus, dorsal subiculum, superior colliculi, peripheral vasculature	Migraine	[15,35]
5-HT_1D_R	G_i/o_	Substantia nigra, basal ganglia, nigrostriatal pathway, hippocampus, raphe nuclei, cortex	Migraine	[15,35]
5-HT_1E_R	G_i/o_	Hippocampus, hypothalamus	Migraine	[15,16]
5-HT_1F_R	G_i/o_	Cortex, hippocampus	Migraine	[15,16]
5-HT_2A_R	G_q/11_	Cortex, vascular smooth muscle	Regulation of vascular tone, mood, cognition, and hallucinogenic effects of various alkaloids	[27,35]
5-HT_2B_R	G_q/11_	Smooth muscle, cardiac tissue, endothelial cells	Cardiovascular function	[28]
5-HT_2C_R	G_q/11_	Gastrointestinal tract, choroid plexus, basal ganglia, substantia nigra, olfactory nucleus, cingulate cortex, lateral habenula, subthalamic nucleus	Gut motility, appetite, cognition, mood, addiction, anxiety, depression, epilepsy, schizophrenia, and obesity	[17,28,35]
5-HT_3_R	Cation channel	Cortex, hippocampus, nucleus accumbens, substantia nigra, ventral tegmental area, area postrema, nucleus tractus solitarius, PNS	Emesis, cognition, anxiety, peristaltic activity, and urinary function	[30]
5-HT_4_R	G_s_ or G_i_	Limbic areas, nucleus accumbens, corpus striatum, globus pallidus, substantia nigra, hippocampus colliculus, cardiac tissue, gastrointestinal tract	Motility, secretion, and visceral sensitivity	[31,35]
5-HT_5_R	G_i/o_	Raphe nuclei, cerebral cortex, hippocampus, amygdala, hypothalamus	Memory, learning, neurotransmitter release, and synaptic plasticity	[33]
5-HT_6_R	G_s_	Cortex, hippocampus, striatum	Learning, memory, addiction, and seizure control	[18,36]
5-HT_7_R	G_s_	Spinal cord, hippocampus, thalamus	Cognitive functions, circadian rhythm, and mood	[24,32]

## 2. 5-HTR Allosteric Modulators

### 2.1. Early Discoveries

**Figure 2:**
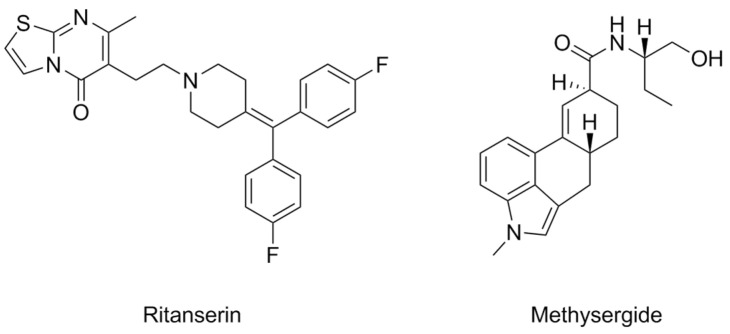
Structural formulas of ritanserin and methysergide.

The earliest mention of allosteric modulators of 5-HTRs dates back to 1987, with 5-HT_2A_R antagonists ritanserin and methysergide [37] (Figure 2). Methysergide is a semisynthetic ergot alkaloid ergometrine derivative, active as a partial agonist of 5-HT_1B_R subtypes and as an antagonist at 5-HT_2A-C_R subtypes, as well as 5-HT_7_R [38,39]. It has been clinically used as a preventative agent for migraines and chronic headaches [38,39,40]. While at first its antimigraine activity was linked to its 5-HT_2A_R antagonist activity, it is currently assumed that it is connected to the activation of 5-HT_1B_ and 5-HT_1D_ receptors [39,40]. However, its lack of selectivity and consequent chronic side effects (mostly fibrotic complications) [41] have meant that methysergide and its congeners ergotamine and dihydroergotamine have been superseded by the triptan class of 5-HT_1B_/_D_R agonists (Figure 1) [40].

Ritanserin was developed as an analog of ketanserin and was described as a competitive antagonist of the 5-HT_2A_R [42,43], only for its mode of action to be later proposed as allosteric [37].

Overall, research involving these two compounds is lacking in several ways: their allosteric mode of action has never been investigated outside of the rat tail artery, and from the point of view of medicinal chemistry, no SAR studies were conducted as follow-up. Moreover, evidence to the contrary was brought to light: their putative allosteric effects may in fact be due to their activity as insurmountable antagonists [44].

### 2.2. Fatty Acid Amides and Cannabinoids

#### 2.2.1. Endocannabinoid Activity at 5-HTRs

**Figure 3:**
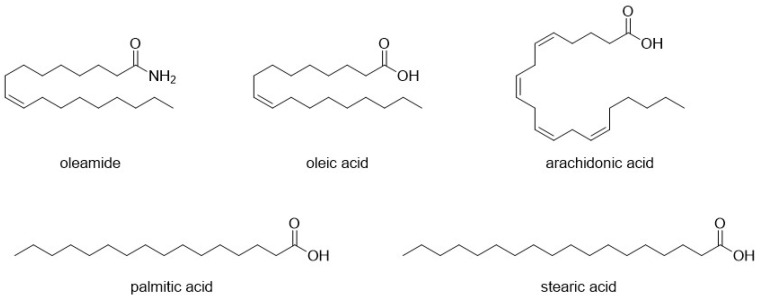
Structural formulas of oleamide and long-chain fatty acids.

Starting from the late 1990s, several studies introduced the role of oleamide (Figure 3) and several other long-chained fatty acid amides as allosteric modulators of 5-HTRs. Oleamide itself is an endogenous neuromodulator that is secreted in the cerebrospinal fluid of humans and other mammals during sleep deprivation, inducing physiological sleep [45]. Its mechanism of action is pleiotropic: outside of its allosteric modulation of the serotonergic system, oleamide also interacts allosterically with GABAergic neurotransmission and the wider endocannabinoid system. In an interesting mirror to its non-canonical interactions with serotonin and GABA receptors, the effect of oleamide on the endocannabinoid system is also indirect, occurring via competition with anandamide and other similar compounds for catabolic enzyme fatty acid amide hydrolase (FAAH) [45].

Fatty acid amides were first identified as positive modulators of mouse 5-HT_2A_R, and 5-HT_2C_R expressed in Xenopus laevis oocytes [46]. These results would later be replicated in rat pituitary tumor P11 cells, where it was found that oleamide did not activate 5-HT_2A_R by itself but strongly potentiated the effect of serotonin at this receptor [47]. Oleamide decreased the response of 5-HT_7_R to serotonin in a way that could not be inhibited by clozapine (a nonselective 5-HT_7_R antagonist), thus suggesting its action as a negative allosteric modulator of 5-HT_7_R in HeLa cells [48]. 

Subsequent radioligand binding experiments evidenced that oleamide caused a 3-fold reduction in the apparent affinity of [3H]5-HT for the 5-HT7R without varying the maximal binding. Oleic acid was also found to be able to bind to 5-HT7R but showed no effect in functional assays. This led the authors to speculate that the lack of the amide function compared to oleamide could make the compound incapable of eliciting changes in 5-HT7R conformation [48].

These results were corroborated by a further study, showing that congeners of oleamide (namely stearic, palmitic, and arachidonic acids) (Figure 3) did not elicit the same effects. Even though the existence of further lipidic amphipathic compounds with activity was hypothesized [49], none were identified in subsequent studies.

#### 2.2.2. Phytocannabinoid Activity at 5-HTRs

Interestingly, the connection between 5-HTRs and the cannabinoid system extends beyond just oleamide. Generally, the canonical effects of endo- and phytocannabinoids are mediated by bespoke cannabinoid receptors such as CB1R, which inhibits the release of several neurotransmitters, including serotonin [50], and CB2R, which modulates the activation of extracellular signal-regulated protein kinases 1 and 2 (ERK1/2), leading to the resolution of acute inflammation [51].

CB1R and 5-HT_2A_R share a relationship much deeper than could be expected: several cannabinoids are modulators of 5-HT_2A_R, and serotonergic dysregulation occurs in mice lacking CB1Rs. This is complemented by the fact that these two receptors are co-expressed in many regions of the brain, especially the neocortex, the hippocampus, and the amygdala, which are linked to memory and learning, as well as emotion [50]. Moreover, serotonin is capable of inducing endocannabinoid release by activating postsynaptic 5-HT_2_Rs. Endocannabinoids then act as retrograde messengers at the presynaptic terminal, reducing glutamate release [52].

**Figure 4:**
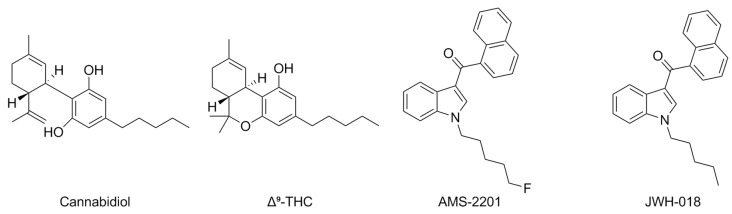
Structures of CBD, THC, AMS-2201, and JWH-018.

Cannabidiol (CBD) (Figure 4) is one of the major components of cannabis, known for its numerous biological activities and rich potential for therapy. Its mechanism of action is pleiotropic, as this compound exerts its effects through several receptors, comprising the canonical CB1R and CB2R, the transient receptor potential vanilloid (TRPV), and the Peroxisome Proliferator-Activated Receptor gamma (PPARγ) [53].

Among its various targets, CBD is also active as an allosteric agonist of 5-HT_1A_R, being able to increase GTP binding to the receptor’s coupled G_i_ protein in Chinese Hamster Ovary (CHO) cells [54]. The activity profile of CBD has been associated in detail with its neuroprotective activity in animal models of hypoxic ischemia, as well as mouse models of hepatic encephalopathy: these effects have also been attributed to 5-HT_1A_R activation, being reverted by antagonists of this receptor [53,55,56,57]. Moreover, CBD has also been shown as a negative allosteric modulator of 5-HT_3_R expressed in Xenopus laevis oocytes (IC_50_ = 0.6 μM) [58,59] and consistently inhibited the Bezold–Jarisch reflex induced by activation of 5-HT_3_R in the rat [60].

The shared chemical space between 5-HTRs and CBRs has also been shown by a recent study demonstrating that the serious adverse effects associated with the recreational use of synthetic cannabinoids AM2201 and JWH-018 (Figure 4) could be associated with their positive allosteric modulation of 5-HT_1A_R. In detail, these two compounds were PAMs of 5-HT_1A_R, enhancing the maximal effect of 5-HT in activating G_i_ by 20.8% and 11.6%, respectively, at a concentration of 10 µM. A similar effect was observed with the 5-HT_1A_R-selective agonist 8-hydroxy-(di-*N*-propylamino)tetralin (8-OH-DPAT). Both AM2201 and JWH-018 significantly inhibited cAMP accumulation. Moreover, AM2201 enhanced the 5-HT-mediated displacement of the 5-HT_1A_R-selective antagonist [^3^H]-WAY100635 in a radioligand binding assay. Crucially, no antagonist displacement was displayed by AMS2201 in the absence of 5-HT. The effects of this compound on dorsal raphe neurons were also evaluated in vivo using CB1R-knockout (CNR1^−/−^) mice, where it enhanced the induction of hypothermia mediated by 8-OH-DPAT while having no effect alone [61].

An important yet underexplored facet of phytocannabinoid activity on serotoninergic receptors resides in their capability to favor or exploit the formation of heteromers between certain 5-HTRs and the two CBRs. The phenomenon of GPCR oligomerization has been proposed since the early 2000s, and it is known to be behind the nuanced activities of many receptors, such as taste receptors T1Rs [62].

In detail, 5-HT_1A_R has been described to form heteromers with CB2R, which might be targeted by CBD [56]. Subsequent studies have shown that the neuroprotective effects of CBD on models of neonatal hypoxia can be attributed to activation of these heteromers [63]. The cognitive impairment induced by Δ^9^-THC (Figure 4) has been associated with the activation of heteromers of 5-HT_2A_R with CB1R [64], which are also expressed in the olfactory neuroepithelium of chronic cannabis users [65]. These findings have recently been corroborated by a study describing the development of novel peptide vectors that selectively disrupt such heteromers, enhancing the antinociceptive effects of THC while avoiding its cognitive side effects [66].

The studies on GPCR heteromerization have seen significant developments in the past few years, and 5-HT_2A_R has been implicated in the formation of several such complexes, as it colocalizes with metabotropic glutamate receptor 2 (in human and mouse frontal cortex), dopamine D2 receptor (in rat striatum), and oxytocin receptor (in rat hippocampus, cingulate cortex, and nucleus accumbens). These heteromers have been proposed as novel targets for pharmacotherapy of autism spectrum disorder [67] and could hold significant promise for the development of novel 5-HT_2A_R allosteric modulators.

### 2.3. Other Natural Substances

Beyond phytocannabinoids, several other natural compounds have been characterized as allosteric modulators of various 5-HTR subtypes.

**Figure 5:**
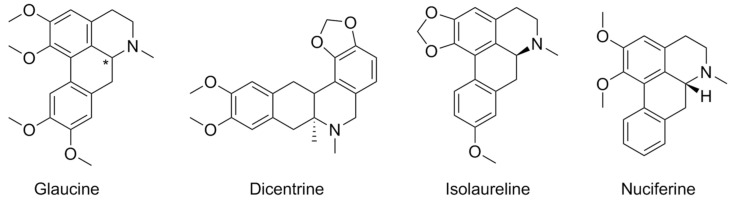
Chemical structures of aporphines glaucine, dicentrine, isolaureline, and nuciferine.

Aporphine alkaloids, a family of compounds known for their varied pharmacological activities, comprise among their ranks several modulators of 5-HTRs. These compounds are usually orthosteric agonists/antagonists of these receptors, and their functional activity depends on their chirality: (*R*)-isomers are agonists, and (*S*)-isomers are active as antagonists. This rule has a few exceptions: both enantiomers of nantenine, roemerine, and nuciferine are antagonists of 5-HT_2A_R. In a recent study, the pharmacological activity in vitro of the enantiomers of related compounds isolaureline, dicentrine, and glaucine was carried out (Figure 5). Both isomers of dicentrine and isolaureline showed similar potencies as orthosteric 5-HT_2A_R antagonists; however, while (*S*)-glaucine acted as a 5-HT_2A_R partial agonist, (*R*)-glaucine appeared to act as a positive allosteric modulator, as it increased 5-HT response by about 25% at 10 µM concentration [68].

Several other natural substances have been reported as negative allosteric modulators of 5-HT_3_R [69]. These include terpene alcohols such as menthol, citronellol, and geraniol, all of which are, however, two to three orders of magnitude less potent than CBD. Ginger constituents were also reported as 5-HT_3_R modulators. In any case, the reported IC_50_ values of these compounds are very high (in the high micromolar to millimolar range), making the question of their in vivo activity somewhat pointless [58].

The most intriguing natural allosteric modulator of 5-HT_3_R is, however, capsaicin. This is a unique alkaloid extracted from chili peppers of the Capsicum family, responsible for the hot, pungent taste of these plants along with dihydrocapsaicin. Both compounds can inhibit h5-HT_3_Rs by interacting at an allosteric site, as demonstrated by radioligand binding assays. Capsaicin’s activity is both time- and concentration-dependent, with IC_50_ values of 62 µM in 5-HT_3A_R-Xenopus oocytes and 54 µM in 5-HT_3A_R-HEK-293 cells. Molecular docking studies performed on 5-HT_3_R modeled on the X-ray structure of the receptor suggested that capsaicin binds to an allosteric transmembrane site located between the transmembrane domains 1, 2, 3, and 4, close to the extracellular domain [70].

### 2.4. Synthetic Allosteric Modulators

#### 2.4.1. Allosteric Modulators of 5-HT_2C_R

5-HT_2C_R has been extensively targeted for the design of allosteric modulators, with the end goal of reaching selective agonists as potential anxiolytics and antiobesity agents. These allosteric modulators are synthetic and were obtained by following various synthetic routes. The original papers provide complete details for the interested reader.

A screening of the Pharmacia (now Pfizer) chemical library led to the identification of PNU-69176E as a PAM of 5-HT_2C_R. The refinement of this compound´s structural features has led to the development of several other allosteric modulators of 5-HT_2C_R, as depicted in Figure 5. PNU-69176E showed a capability to increase [^3^H]-5-HT binding in 5-HT_2C_R-transfected CHO cells at an optimum concentration of 25 μM, while higher concentrations disrupted membrane structures and inhibited radioligand binding. From a functional point of view, PNU-69176E at a concentration of 10 µM can decrease the EC_50_ of 5-HT from 27 to 10 nm while increasing its E_max_ by 20%. The binding of the antagonist [^3^H]-mesulergine in 5-HT_2C_R-transfected CHO cells was not affected by PNU-69176E. In addition, this compound was also capable of slowly inducing conformational changes in the whole receptor population that led to constitutive activation. From a structural point of view, the activity of PNU-69176E was linked to its amphipathic nature, which it has in common with amphipathic lipids that act as allosteric 5-HTR modulators. This is indeed an important observation, as the bulky alkyl moiety may anchor the compound to the cell membrane, facilitating the interaction of the polar groups in the α-D-galactopyranoside moiety with allosteric sites close to the membrane itself [71]. Research interest in PNU-69176E languished for several years until a follow-up study demonstrated an optimized synthetic route for its obtainment starting from commercial picolinic acid. This study also confirmed its efficacy and potency as a positive allosteric modulator of 5-HT_2C_R and determined its selectivity over 5-HT_2A_R. While a diastereomer of PNU-69176E was additionally obtained through this synthetic route, it was inactive at 5-HT_2C_R and 5-HT_2A_R [72].

The complex amphipathic scaffold of PNU-69176E compelled researchers to look for simplified derivatives. As the binding site for this compound was yet unknown, the authors first observed that its structure contained three distinct regions that could be important for binding, namely the lipophilic undecyl tail, the piperidine carboxamide core, and the sugar moiety. This latter part was the first point of structural exploration, being replaced with different amino alcohols that could emulate its capability to form hydrogen bonds, with hydroxy groups situated at a distance of two carbon atoms from the amide linker. Additionally, the undecyl tail was replaced in some cases with more rigid cyclohexyl or cyclohexylethyl chains.

**Figure 6:**
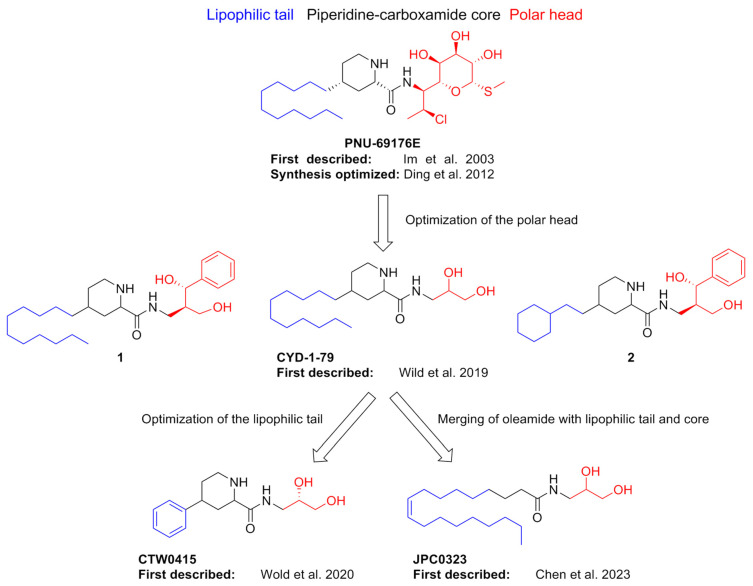
Development of PNU-69176E and related derivatives over the past twenty years [71,72,73,74,75].

These compounds were tested in a Ca^2+^ release assay in CHO cells stably transfected with human 5-HT_2C_R in the presence of 5-HT. From a first screening at 1 nM, none of the tested compounds mediated Ca^2+^ release independently of 5-HT; however, most of them also had no significant activity as allosteric modulators. Compounds **1** and CYD-1-79 (Figure 6) were exceptions: the former was active as a NAM, while the latter showed activity as a PAM, increasing 5-HT E_max_ by about 20%, similarly to PNU-69176E but having no effect on its EC_50_. Interestingly, the difference between the two compounds is one additional carbon in the spacer. Changing the lipophilic tail of **1** with a cyclohexylethyl moiety resulted in **2**, which was active as a weak NAM, while all other modifications regarding this portion of the scaffold were ineffective at affording allosteric modulators. The stereochemistry of the 1,2-diol fragment of CYD-1-79 was also essential for PAM activity, with the *S*-configuration being preferred. The authors also showed that CYD-1-79 was selective for 5-HT_2C_R, demonstrating no intrinsic activity at either 5-HT_2A_R or 5-HT_2B_R and showing weak or no displacement of the specific radioligand from other 5-HT receptors, dopamine receptors, and monoamine transporters. The recent disclosure of the 5-HT_2C_R structure also enabled the authors to carry out the very first allosteric 5-HT_2C_R docking study, which highlighted a possible binding site close to the orthosteric one. In vivo studies were carried out in rat models, proving that CYD-1-79 had a good pharmacokinetic profile (half-life after iv administration of 5 mg/kg was 6.59 h) while being effective in suppressing spontaneous ambulation as well as in attenuating cocaine cue reactivity in rats [73].

The optimization of the undecyl lipophilic tail was the focus of subsequent work by the same group, whose ultimate goal was to achieve smaller and more rigid molecules. These compounds were assayed similarly to the original series of PNU-69176E analogs. The optimization of the structure of CYD-1-79 led to several compounds endowed with similar activity at a concentration of 1 nM. Compound CTW0415 (Figure 6) emerged as the most significant PAM and exhibited optimal pharmacokinetics and physicochemical properties, combined with a very selective off-target profile. Compared to CYD-1-79, it had slightly enhanced efficacy, raising 5-HT E_max_ to 127%. Molecular docking studies were also carried out, corroborating the existence of the unique allosteric pocket of 5-HT_2C_R [74].

The most recent developments in this line of research have seen the hybridization of the polar moiety of these compounds with a lipophilic tail derived from oleamide, thus combining two pharmacophoric groups of two known 5-HTR allosteric modulators. This approach also had the significant advantage of reducing the chemical complexity of the target compounds, as well as improving their drug-likeness. Several compounds of this series showed significant efficacy at 1 nM in improving 5-HT-mediated calcium efflux. Interestingly, while some of them were selective PAMs of 5-HT_2C_R, others were described as dual 5-HT_2A_R/5-HT_2C_R PAMs. None of these compounds were reported as PAMs of 5-HT_2B_R. A full characterization was conducted for dual PAM JPC0323 (Figure 6), which evoked a 44% increase in maximum 5-HT-induced Ca^2+^ intake and also showed negligible displacement at orthosteric binding sites of a number of GPCRs and transporters and exhibited favorable pharmacokinetic parameters. In rats, JPC0323 suppressed spontaneous ambulation in a 5-HT_2C_R-dependent manner, suggesting that the compound has a preference for 5-HT_2C_R over 5-HT_2A_R [75].

**Figure 7:**
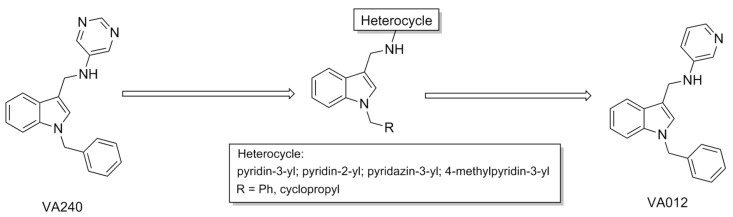
VA240 development.

The pace of research in the medicinal chemistry of 5-HT_2C_R allosteric modulators has significantly increased over the past few years. Similarly to PNU-69176E, compound VA240 was identified as a moderate PAM of 5-HT_2C_R (potentiating the effect of 5-HT with E_max_ = 20%) after high-throughput screening of a chemical library from Vivia Biotech. After its identification, the authors synthesized two series of analogs by modifying either the pyrimidine or the phenyl ring attached to the central indole scaffold of VA240 (Figure 7). While most of these compounds were completely inactive, modification of the pyrimidine afforded the best results, with 3-pyridyl analog VA012 exhibiting the most significant effect (35% at 10 μM). The potency of VA012 was also quantified (EC_50_ = 16 nM), and its lack of intrinsic activity at 5-HT_2C_R was confirmed. VA012 is selective over the other 5-HT_2_R subtypes and a set of off-target GPCRs. The allosteric nature of VA012 binding to 5-HT_2C_R was corroborated by the low displacement of the orthosteric ligands 5-HT, mesulergine, and clozapine from 5-HT_2C_R. After administration i.p. in the rat (10 mg/kg), VA012 exhibited a brain-to-plasma ratio of 3.8 after 120 min; therefore, it was suitable for evaluation in animal models of obesity. VA012 was highly active in rodent feeding models, reducing both food intake and body weight gain. However, the compound induced hyperlocomotion in two behavioral tests, suggesting that such side effects might limit its therapeutic utility, at least at certain doses [76].

**Figure 8:**
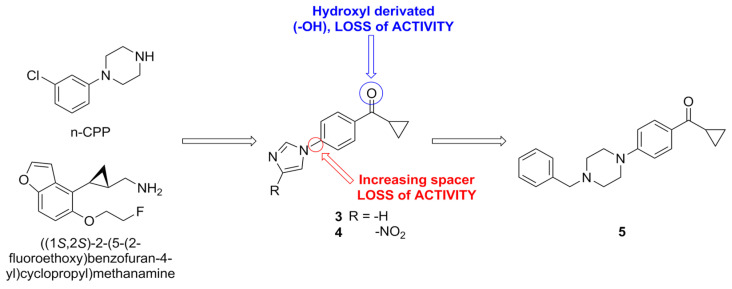
Development overview of azo-linked compounds.

In another recent work, the structures of meta-chlorophenylpiperazine and a 2-phenylcyclopropylmethylamine derivative, two known 5-HT_2C_R agonists with favorable pharmacodynamic and pharmacokinetic properties, were used as inspiration for the synthesis of a new series of compounds featuring a phenylcyclopropylmethane or phenylcyclopropylmethanone scaffold. This led to the discovery of two imidazole derivatives of the phenylcyclopropylmethanone series (compounds **3** and **4**, Figure 8), which were active as PAMs of 5-HT_2C_R but were non-selective and displayed a similar activity profile at 5-HT_2B_R. Interestingly, their respective methanol derivatives lost their PAM 5-HT_2C_R activity. Compounds **3** and **4** were the basis for the synthesis of further derivatives of the phenylcyclopropyl methanone class, substituting the imidazole with other heterobicyclic scaffolds or with a piperazine unit. One of the piperazine derivatives, (compound **5**, Figure 8) showed activity as a 5-HT_2C_R PAM, increasing 5-HT E_max_ to 150% at a concentration of 10 µM, as well as a 5-HT_2B_R NAM, decreasing 5-HT EC_50_ tenfold. All modifications of the scaffold of compound **5**, which involved the introduction of bulky substituents on the basic piperazine nitrogen, resulted in a complete loss of PAM activity at 5-HT_2C_R. Similarly, the introduction of a spacer between the phenylcyclopropyl methanone scaffold of **3** and the imidazole resulted in a complete loss of PAM activity. The 5-HT_2B_R NAM activity of compound **5** was described as promising for the enhancement of the therapeutic window of non-selective orthosteric 5-HT_2_R agonists.

**Figure 9:**
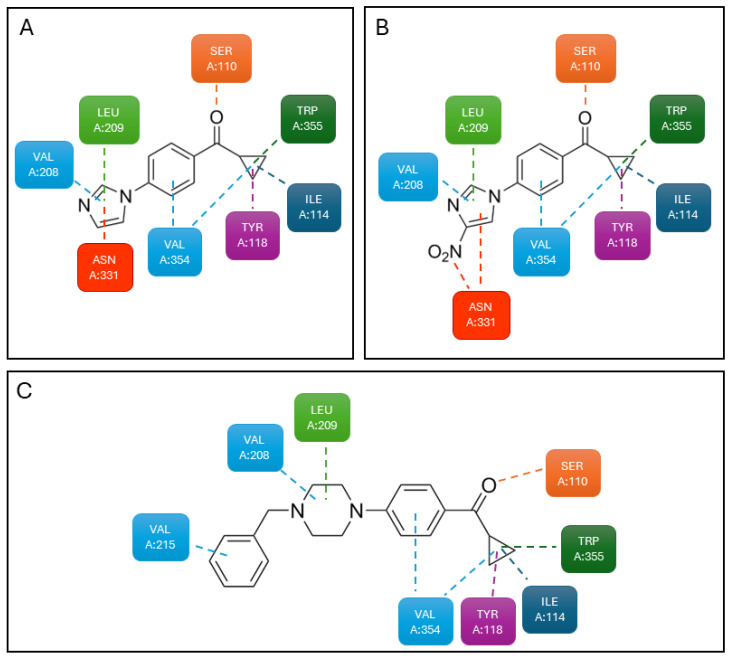
(**A**) Interactions of **3** with 5-HT_2C_R; (**B**) interactions of **4** with 5-HT_2C_R; and (**C**) interactions of compound **5** with 5-HT_2C_R. Adapted from [77].

Docking studies were carried out, further supporting the binding of these compounds to an extracellular allosteric site of 5-HT_2C_R. Compounds **3** and **4** formed very similar, mostly hydrophobic interactions between the imidazole core and residues in the extracellular loop EL2 and between the terminal cyclopropyl and other hydrophobic residues. The bridging carbonyl group formed a hydrogen bond with a serine residue on transmembrane helix 2 (Figure 9A). Compound **4** formed additional hydrogen bonds with an asparagine residue on transmembrane helix 4 by virtue of its nitro group in position 4 of the imidazole ring (Figure 9B). Compound **5** behaved similarly to compounds **3** and **4**, but the additional interactions caused by the benzylpiperazine moiety increased its binding energy to the receptor (Figure 9C).

Finally, the in vivo effects of compound **5** were evaluated via intracerebroventricular administration in the rat. The compound suppressed food intake after 3 and 6 h from administration, in a similar manner as the reference antiobesity drug lorcaserin. The authors speculated that since compound **5** did not exhibit any effect on cumulative food intake after 24 h, it was most likely metabolized. However, the pharmacokinetic profile of this compound was not disclosed [77].

**Figure 10:**
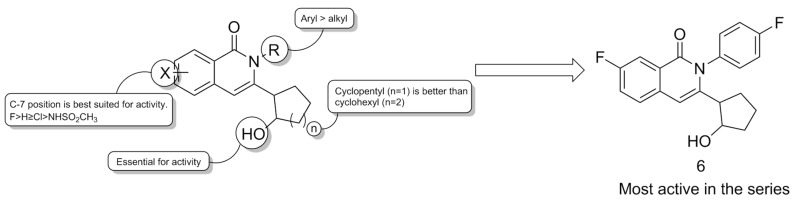
SAR of isoquinolin-1(*2H*)-ones and structural formula of compound **6**. Adapted from [78].

To date, only one research paper has been published regarding the development of 5-HT_2C_R PAAMs. This strategy was prompted by the withdrawal of the antiobesity drug lorcaserin from the market, which led the authors to switch away from orthosteric agonists. A virtual screening campaign afforded a series of compounds with an isoquinolin-1-one scaffold, which was capable of docking into the ergotamine (orthosteric) and the tripeptide (allosteric) binding sites of 5-HT_2C_R. The synthesis of these compounds, as well as of their isoindolin-1-one analogs, allowed the authors to sketch some structure–activity relationships after their evaluation via a luciferase assay in HEK293T cells. The original isoquinolin-1-one scaffold afforded the best results, especially when linked to an aryl on the nitrogen atom, a 1-hydroxycyclopentyl moiety on C-3, and a fluorine atom on C-7 (Figure 10). The hydroxy group of the hydroxycyclopentyl moiety proved to be essential for the activity of the compounds. The most promising of these compounds, **6** (Figure 10), enhanced 5-HT potency by 3 times, with an EC_50_¬ in the low nanomolar range. Thus, compound 6 was selected for further in vivo studies based on its relative selectivity towards the other 5-HT_2_R subtypes. Compound **6** showed decent pharmacokinetic properties, quickly reaching the brain, maintaining a 1:1 brain/plasma ratio, and showing good stability in Balb/c mice brain homogenate. Moreover, studies conducted on Sprague Dawley rats showed that compound 6 was capable of significantly reducing food intake after 3 days from treatment with a 50 mg/kg dose, with comparable results to lorcaserin (5 days from treatment at 10 mg/kg) [78].

An overview of the 5-HT_2C_R allosteric modulators discussed in this section is provided in Table 2.

#### 2.4.2. Allosteric Modulators of 5-HT_3_R

**Figure 11:**
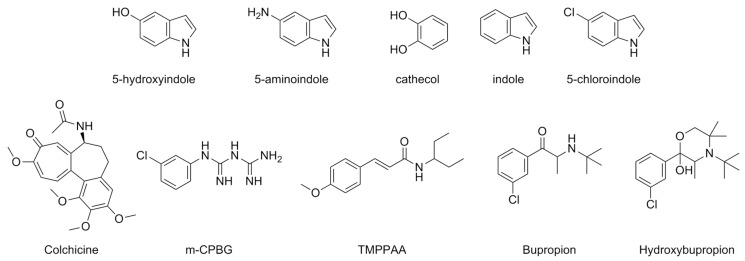
Structural formulas of allosteric modulators of 5-HT_3_.

The exploration of allosteric modulators of 5-HT_3_R can be traced back to 1994 when the efficacy of 5-hydroxyindole (5-HI) (Figure 11) as a PAM of this receptor was reported in mouse N1E-115 neuroblastoma cells [79]. Subsequent research by Van Hooft et al. further corroborated the activity of 5-HI and of some of its analogs (5-aminoindole, catechol, and indole) (Figure 11) in the same model [80]. However, despite the promising indications regarding these compounds’ potential as allosteric modulators, their effectiveness was observed at very high concentrations, in the millimolar range [79,80]. Moreover, 5-HI demonstrated no selectivity at 5-HT_3_R as it can also interact with nicotinic α7 receptors [81]. In a recent study, another analog of 5-HI, 5-chloroindole (Cl-indole), exhibited activity as a potent and selective 5-HT_3_R PAM. Cl-indole can enhance by about 30% the activity of both 5-HT and several other well-known 5-HT_3_R agonists in HEK293 cells, stably expressing the human 5-HT_3A_R subunit. This activity was confirmed through intracellular calcium assays, voltage clamp, and radioligand binding studies, all of which indicated that Cl-indole binds to an allosteric site. Moreover, investigation of 5-HT_3_R-mediated contraction of the mouse urinary bladder showed that Cl-indole is also active as a PAM of native 5-HT_3_Rs. Indeed, while Cl-indole alone had no effect on the amplitude of contraction, co-administration with the 5-HT_3_R agonist *meta*-Chlorophenyl biguanide (mCPBG) greatly increased peak amplitude [82].

Colchicine is another allosteric modulator of the 5-HT_3_ receptor (Figure 11). This compound is a well-known microtubule-depolymerizing agent that acts as a competitive antagonist of GABA-A and Glycine receptors. Colchicine’s activity was evaluated in Xenopus laevis oocytes expressing mouse or human 5-HT_3_R (m5-HT_3A_R and h5-HT_3A_R, respectively). Interestingly, colchicine demonstrates distinct effects on these receptors, as it acts as a NAM in m5-HT_3A_Rs, while, in h5-HT_3A_Rs, it behaves as a PAM at low 5-HT concentrations and as a NAM at high 5-HT concentrations, although both phases of its activity have not been quantified. Additionally, radioligand binding assays have confirmed the allosteric modulator nature of colchicine. Its unique interaction profile makes colchicine a valuable pharmacological tool for investigating interspecies differences, particularly provided the high sequence homology (>80%) between m5-HT_3A_R and h5-HT_3A_R [83].

mCPBG (Figure 11) is a 5-HT_3_R agonist that can behave as a PAM of heteromeric 5-HT_3AB_ receptors. A combination of experimental and computational studies elucidated that 5-HT binds in a binding pocket located at the interface between two A subunits. This evidence was confirmed by the differences in 5-HT potency between 5-HT_3A_R and 5-HT_3AB_R. Indeed, the EC_50_ for the homomeric 5-HT_3A_R is 3.4 μM, while for the heteromeric 5-HT_3AB_R, it is 24.1 μM. However, the data for mCPBG differ, as it maintains a similar EC_50_ for both the homomeric isoform 5-HT_3A_R (3.8 μM) and the heteromeric isoform 5-HT_3AB_R (2.8 μM), suggesting that it does not bind to the agonist binding pocket but interacts with an allosteric site. This was further supported by mutagenesis experiments confirming mCPBG’s ability to interact with the heteromeric receptor. Additionally, mCPBG can enhance ion currents mediated by 5-HT upon co-administration [84].

In 2014, Gasiorek et al. elucidated the unique activity of trans-3-(4-methoxyphenyl)-N-(pentan-3-yl)acrylamide (TMPPAA) (Figure 11), previously developed by the same group. The study demonstrated that TMPPAA could function both as a positive allosteric modulator (PAM) and an allosteric agonist, as it not only enhances 5-HT activity by about 30% but also activates the receptor itself. The investigation involved various cell lines and employed electrophysiological techniques, fluorescence, and radioligand binding assays. TMPPAA interacts with a specific site in the transmembrane domain, which distinguishes it from other allosteric modulators that do not bind to this site. The study suggested that this allosteric site may interact with the orthosteric site, resulting in the compound’s intrinsic agonist activity [85].

Bupropion (Figure 11), an FDA-approved drug for treating depression and aiding smoking cessation, exerts its effects through various mechanisms. It inhibits dopamine and norepinephrine reuptake transporters and also targets nicotinic acetylcholine receptors, which belong to the Cys-loop superfamily of ion channels, similar to 5-HT_3_R. Additionally, bupropion functions as a negative allosteric modulator (NAM) of 5-HT_3A_Rs. It can reversibly block inward currents of 5-HT_3A_Rs expressed in Xenopus laevis oocytes in a concentration-dependent manner, with an inhibitory potency of 87 μM. Furthermore, studies have examined the effects of its primary metabolite, hydroxybupropion, which also acts as a NAM with a similar IC_50_, confirmed through radioligand binding assays. An intriguing aspect of bupropion’s activity at 5-HT_3A_Rs is its non-use-dependent nature. Even when cells were pre-incubated with the drug, no discernible differences were observed compared to experiments without pre-incubation. This characteristic of bupropion (and its metabolite) could provide new insights into treating depression or anxiety disorders, as 5-HT_3_R antagonists have shown promise in such conditions [86].

An overview of these allosteric modulators of 5-HT_3_R is provided in Table 3.

## 3. Conclusions and Future Perspectives

Until recently, high-throughput screening of large chemical libraries was the primary strategy for discovering allosteric modulators. However, the recent outbursts of crystallography and cryoelectron microscopy (cryo-EM) studies have provided details on the structures of several GPCRs bound to allosteric modulators, opening novel opportunities for the structure-based discovery of allosteric modulators [87,88].

To date, the structures of all but 5-HT_5B_R have been elucidated via X-ray crystallography or cryo-EM [89,90]. Yet, none of these structures has been used for the structure-based design of 5-HTR allosteric modulators. It is expected that such receptor protein structural insight will be exploited to discover novel 5-HTR allosteric modulators. In fact, the computational docking of small molecules to the surface of GPCR structures has already served to identify potential allosteric sites. To this end, Hedderich et al. have docked small molecular probes to 557 GPCR structures and predicted various previously uncharacterized allosteric sites [91]. Interestingly, the study did not include 5-HTRs, thus leaving room for discovering unexploited sources for designing 5-HTR allosteric modulators.

Peptides and proteins are stepping into the spotlight as a source for identifying allosteric modulators [92]. In this respect, the GPCR interactome (which includes transducers, accessory proteins, pepducins, and extracellular and transmembrane domains) represents an underexplored space for the discovery of allosteric modulators that might become boundless if complemented by nanobodies or conotoxins. Yet, peptides feature distinct pros (high bioactivity and tolerability) and cons (relatively poor pharmacokinetic properties) over small molecules that can be overcome by moving towards peptidomimetics, which combines the advantages of both peptides and small molecules by mimicking the chemical features responsible for bioactivity and higher chances of drug-likeness.

Indeed, it has been known for quite a while that peptides such as 5-HT-moduline are capable of interacting with 5-HT_1B_R via allosteric sites, eliciting stress and anxiety in a mouse model [93,94]. Similarly, it was found that a peptide whose structure was based on the fourth fragment of the third intracellular loop of 5-HT_2C_R was capable of allosterically potentiating 5-HT_2C_R signaling in CHO cells as well as in Sprague Dawley rats, contributing to enhancing the anti-impulsive effects of known agonist WAY163909, as well as augmenting the hypolocomotive activity of 5-HT_2C_R agonists [95].

Dualsteric ligands might be considered as a particular subgroup of allosteric modulators. Dualsteric modulators are bivalent (or bitopic) as they feature two pharmacophores; one binds the protein at the orthosteric and the other at an allosteric site [96]. This type of ligand has recently gained attention as they should combine, at least in theory, high affinity (via the orthosteric site) with high selectivity (via an allosteric site). Concerning monoamine receptors, several examples of dualsteric ligands targeting dopamine D2 or D3 receptors and adrenergic receptors have been reported to date (see, for example, references [97,98]). A fragment-linking methodology was recently proposed to achieve dualsteric ligands with enhanced selectivity toward specific GPCRs, combining moieties that bind the conserved orthosteric sites with moieties that selectively attack the more specific allosteric pockets. This methodology was also applied to 5-HT_1B_R and 5-HT_2B_R, leading to two very selective compounds, and showing significant promise for the future design of compounds with specific multi-target profiles and fewer undesired effects [99].

In conclusion, more than forty years after the first 5-HTR-subtype-selective ligand was discovered [100], tremendous efforts have been made to find selective agonists and antagonists for each of the 5-HTR subtypes. Nevertheless, medicinal chemists still have room to develop novel chemical entities that target 5-HTRs.

## Figures and Tables

**Table 2:** Overview of main synthetic allosteric modulators of 5-HT_2C_R and their effects in animal models. ^a^: not tested. CHO: Chinese Hamster Ovary cell line. HEK293: Human Embryo Kidney cell line.

Compound	Type of Modulator	Cell Model	Animal Model	Effects on Animal Model	Reference
PNU-69176E	PAM (PAM-agonist?). It induces slow conformational changes in the receptor population leading to constitutive activation.	CHO	N.T. ^a^	N.T. ^a^	[72]
**1**	NAM	CHO	N.T. ^a^	N.T.^a^	[73]
CYD-1-79	PAM	CHO	Sprague Dawley rats	Suppression of spontaneous ambulations; attenuation of cocaine cue reactivity	
**2**	Mild NAM	CHO	N.T. ^a^	N.T. ^a^	
CTW0415	PAM	CHO	N.T. ^a^	N.T. ^a^	[74]
JPC0323	Dual PAM (5-HT_2C_R/5-HT_2A_R)	CHO	Sprague Dawley rats	Suppression of spontaneous ambulations	[75]
VA240	PAM	CHO	N.T. ^a^	N.T. ^a^	[76]
VA012	PAM	CHO	Wistar rats	Reduction in food intake; reduction in weight gain; hyperlocomotion (side effect)	
**3**	Dual PAM (5-HT_2C_R/5-HT_2A_R)	HEK293T cells	N.T. ^a^	N.T. ^a^	[77]
**4**	Dual PAM (5-HT_2C_R/5-HT_2A_R)	HEK293T cells	N.T. ^a^	N.T. ^a^	
**5**	PAM (5-HT_2C_R) NAM (5-HT_2B_R)	HEK293T cells	Sprague Dawley rats	Reduction in food intake	
**6**	PAAM	HEK293T cells	Sprague Dawley rats	Reduction in food intake	[78]

**Table 3:** Overview of synthetic allosteric modulators of 5-HT_3_R. N1E-115: Mouse Neuroblastoma cell line. HEK293: Human Embryo Kidney cell line. COS-7: CV1 simian kidney cells carrying SV40 viral genetic material.

Compound	Type of Modulator		Cell Model	Reference
5-Hydroxyindole (5-HI)	PAM		N1E-115	[80]
5-Aminoindole	PAM		N1E-115	
Cathecol	PAM		N1E-115	
Indole	PAM		N1E-115	
5-Chloroindole (Cl-indole)	PAM		HEK293	[82]
Colchicine	m5-HT_3_ARs: NAM		Xenopus laevis oocytes	[83]
	h5-HT_3_ARs:	-PAM low concentrations -NAM high concentrations		
*Meta*-chlorophenylbiguanide (mCPBG)	PAM		Xenopus laevis oocytes	[84]
Trans-3-(4-methoxyphenyl)-*N*-(pentan-3-yl)acrylamide (TMPPAA)	PAM		COS-7 Xenopus laevis oocytes HEK293	[85]
Bupropion	NAM		Xenopus laevis oocytes	[86]
Hydroxybrupropion	NAM		Xenopus laevis oocytes	

## Data Availability

Data sharing is not applicable.
